# Effect of diurnal temperature range on emergency room visits for acute upper respiratory tract infections

**DOI:** 10.1186/s12199-021-00974-w

**Published:** 2021-05-03

**Authors:** Jin Young Jang, Byung Chul Chun

**Affiliations:** 1 Department of Public Health, Korea University Graduate School, Seoul, Republic of Korea; 2 Department of Preventive Medicine, Korea University College of Medicine, Seoul, Republic of Korea

**Keywords:** Acute upper respiratory tract infections, Diurnal temperature range, Time-series, Climate factors

## Abstract

**Background:**

An acute upper respiratory tract infection (URI) is the most common disease worldwide, irrespective of age or sex. This study aimed to evaluate the short-term effect of diurnal temperature range (DTR) on emergency room (ER) visits for URI in Seoul, Korea, between 2009 and 2013.

**Methods:**

Daily ER visits for URI were selected from the National Emergency Department Information System, which is a nationwide daily reporting system for ER visits in Korea. URI cases were defined according to *International Classification of Diseases, 10*^*th*^ *Revision* codes J00–J06. The search for DTR effects associated with URI was performed using a semi-parametric generalized additive model approach with log link.

**Results:**

There were 529,527 ER visits for URI during the study period, with a daily mean of 290 visits (range, 74–1942 visits). The mean daily DTR was 8.05 °C (range, 1.1–17.6 °C). The cumulative day (lag 02) effect of DTR above 6.57 °C per 1 °C increment was associated with a 1.42% (95% confidence interval [CI] 0.04–2.82) increase in total URI. Children (≤ 5 years of age) were affected by DTR above 6.57 °C per 1 °C, with 1.45% (95% CI 0.32–2.60) at lag 02, adults (19–64 years) with 2.77% (95% CI 0.39–5.20) at lag 07. When the DTR (lag02) was 6.57 °C to 11.03 °C, the relative risk was significant at 6.01% (95% CI 2.45–9.69) for every 1 °C increase in youth subjects aged for 6 to 18 years.

**Conclusions:**

DTR was associated with a higher risk for ER visits for URI. In addition, the results suggested that the lag effects and relative risks of DTR on URI were quite different according to age.

**Supplementary Information:**

The online version contains supplementary material available at 10.1186/s12199-021-00974-w.

## Introduction

Acute upper respiratory tract infections (URI), which include nasopharyngitis (common cold), sinusitis, pharyngitis, tonsillitis, tracheitis, and epiglottitis, are the most common diseases worldwide, irrespective of age or sex [1]. URI are caused by a wide variety of viruses and bacteria [2]. The incidence of the common cold ranges from 6 to 10 episodes per year in preschool-age children, 7 to 12 in children in elementary school and, in adolescents and adults, from two to four episodes [2, 3]. Symptoms of URI commonly include cough, nasal congestion, and discharge, facial pain, sneezing, fever, headache, myalgia, and malaise [4]. These symptoms usually resolve after approximately 1 week. However, URI sometimes predispose to complications such as pneumonia and are the leading cause of economic burden on healthcare resources, as well as absences from work and/or school [3].

Seasonal variation in the incidence of URI is a common phenomenon, there is a widely held folklore that rapid changes in air temperatures are the cause of URI epidemics [5]. Diurnal temperature range (DTR) generally is defined as the difference between maximum and minimum temperatures within a single day. In previous studies, a 1 °C decrease in temperature increased the estimated risk for URI incidence [6, 7]. The occurrence of URI increases in response to every 1 °C increase in DTR [8–10]. In addition, recent studies in Korea proposed that respiratory diseases are affected by increases in DTR [11–13]. There is also a study that analyzed the years of life lost (YLL) among health effects related to DTR [14]. As far as we know, no previous study has reported an association between DTR and daily emergency room (ER) visits for URI.

The present study aimed to evaluate the short-term effect of DTR on ER visits for URI in Seoul, Korea, between 2009 and 2013. We sought to examine the difference in the effect of DTR between groups by stratification analysis by gender and age.

## Materials and methods

### Study area

Seoul (37° 25′-37° 41′ N, 126° 45′-127° 11′ E) is located in the northwest of the Korean peninsula and is approximately halved into northern and southern regions by the Han River. In 2011, it had an area of 605.21 km^2^ and has a gross east-west distance of 36.78 km and 30.30 km south-north. In the same year, the city’s total population was 10,528,774, of which 9.97% was ≥ 65 and 4.06% were ≤ 5 years of age [15]. The climate is intermediate between continental and oceanic. In the winter, a seasonal wind from the northwest results in dry and low temperatures, while in the summer, it is usually very hot and humid resulting from the effects of subtropical oceanic air currents.

### Study population

Data regarding daily emergency room (ER) visits for acute upper respiratory tract infections (URI) between January 1, 2009, and December 31, 2013 were collected from the National Emergency Department Information System (NEDIS). The NEDIS is a real-time reporting system for ER visits in Korea. The coverage rate for all hospitals in Seoul was 58% in 2009, 58% in 2010, 59% in 2011, 60% in 2012, and 81% in 2013 [16].

URI cases were defined as patients assigned discharge diagnostic codes J00 to J06 from the International Classification of Diseases, 10th Revision (ICD-10). ER visits for URI were defined as the number of ER visits for URI divided by the mid-year population per 1000 for each year.

The dataset contained no personally identifiable patient information. Dependent variables in the analysis included date of visit and patient age and sex. This study analyzed publicly available data sets and was, therefore, exempt from institutional review board approval and requirements for informed consent.

### Environmental data

Daily mean climate was calculated for average temperature (°C), DTR (°C), and relative humidity (%), in Seoul between December 1, 2008, and December 31, 2013. Local climate data were obtained from the Korea Climate Administration and were measured at a fixed-site station (37° 34′ N, 126° 57′ E [elevation, 85.8 m]) [17].

Estimates of daily mean ambient concentrations of air pollution were obtained from the National Ambient Air Quality Monitoring Information System maintained by the Ministry of Environment, which collects data on air pollution levels through automated fixed-site monitoring stations [18]. Air pollutant data were obtained from 25 observation stations in Seoul between December 1, 2008, and December 31, 2013. Independent variables included in the analysis were particulate matter of median aerometric diameter < 10 microns (PM_10_ [μg/m^3^]) and ozone (O_3_ [in parts per billion (ppb)]) measured at each station.

### Statistical analysis

From 2009 to 2013, the distribution of daily ER visits for URI and environmental data are presented in a time-series plot. We explored the descriptive statistics of daily URI cases, climate, and air pollutant variables and performed Spearman’s correlation analysis between URI cases and the exposure variables. The lag structures consisted of the event day and the seven previous days. We used the semi-parametric generalized additive model (GAM) approach with log link to explore the associations between daily DTR and ER visits for URI [19].

The basic model is as follows:

$$ \log \left[\mathrm{E}\left(\mathrm{Yt}\right)\right]=\upalpha +\mathrm{s}\left(\mathrm{DTR}\right)+\mathrm{s}\left(\mathrm{average}\ \mathrm{temperature}\right)+\mathrm{s}\left(\mathrm{relative}\ \mathrm{humidity}\right)+\mathrm{s}\left(\mathrm{PM}10\right)+\mathrm{s}\left(\mathrm{O}3\right)+\mathrm{time}+\mathrm{DOW}+\mathrm{Holiday}+\kern0.5em \mathrm{s} in\frac{2\pi t}{T}+\mathit{\cos}\frac{2\pi t}{T} $$

in which *t* refers to the day of the observation; E(Yt) denotes the estimated daily ER visits for URI on day *t*; *α* is the intercept; **s**( ) denotes a thin plate regression spline function for nonlinear variables; time is days of calendar time on day *t*; *T* is the length of seasonal cycle, 365 days; DOW is the day of the week; and Holiday denotes public holidays.

Potential confounders, such as long-term trend of time and seasonality, were controlled for using time term and Fourier terms, respectively [20]. Climate factors and air pollutants variables were controlled for using thin plate regression splines as a smoothing function of the GAM. DOW and Holiday were adjusted as dummy variables in the model. We used the negative binomial model because of overdispersion of the data [21].

Stratified analyses were performed to examine whether associations between DTR and ER visits for URI were similar across age and sex. Residuals of the basic models were used to check whether there were discernible patterns and autocorrelation by means of residual plots.

The best model is the one with the smallest Akaike information criterion (AIC) value. All statistical analyses and plot constructions were performed using the *mgcv* package and *gamRR* package of the R software version 3.6.1 (R foundation for Statistical Computing, Vienna, Austria).

## Results

There were 529,527 ER visits for URI between 2009 and 2013 (Table 1); the mean number of ER visits for URI per day during the same period was 290.02 (range, 74–1,942 visits) (Table 2). The number of ER visits for URI was the highest in 2009 (0.032 ± 0.026 per 1000 persons) and 2010 (0.032 ± 0.018 per 1000 persons) and the lowest in 2011 (0.025 ± 0.015 per 1000 persons).

**Table 1:** Daily emergency room visits for acute upper respiratory tract infections according to several characteristics in Seoul, Korea, 2009 to 2013 (*n* = 529,527)

Characteristic	Cases, *n*	%
Sex		
Male	278,777	52.7
Female	250,750	47.3
Age, years		
0–5	297,364	56.2
6–18	74,337	14.0
19–64	147,451	27.8
≥ 65	10,375	2.0
Year		
2009	119,137	22.5
2010	119,104	22.5
2011	92,388	17.4
2012	104,295	19.7
2013	94,603	17.9
Day of the week		
Monday	65,777	12.4
Tuesday	58,966	11.1
Wednesday	57,394	10.9
Thursday	57,818	10.9
Friday	57,770	10.9
Saturday	84,552	16.0
Sunday	147,250	27.8
Public holidays		
Holiday	262,001	49.5
Non-holiday	267,526	50.5

**Table 2:** Summary statistics of daily emergency room visits for acute upper respiratory tract infections, and climate factors and air pollutants in Seoul, Korea, 2009 to 2013

Daily data	Mean	SD	Min	P25	P50	P75	Max
Upper respiratory tract infections	290.02	193.29	74	174	227	331.5	1942
Climate factors							
Diurnal temperature range (°C)	8.05	2.75	1.10	6.10	8.10	10	17.6
Average temperature (°C)	12.41	11.03	−14.5	3.3	13.9	22.7	31.8
Relative humidity (%)	60.14	15.12	20	49	60	71	97
Air pollutants							
PM_10_ (μg/m^3^)	47.14	28.74	4.96	28.59	41.83	57.46	298
Ozone (parts per billion)	20.6	11.1	1.8	11.7	19.3	28.0	71.1

*SD* standard deviation, *Min* minimum, *P* percentile, *Max* maximum, *PM*_*10*_ particulate matter with a median aerometric diameter <10 microns

In particular, a remarkably high rate of URI was observed among the 0–5-year age group (56.2%). The proportion of URI cases on public holidays (49.5%), including Saturday and Sunday, was similar to that on non-holidays (50.5%). Public holidays were 110 days in 2009, 113 days in 2010, and 116 days from 2011 to 2013, respectively. Furthermore, the total number of URI cases on Sunday (27.8%) was highest compared with any other day of week, followed by Saturday (16%).

During the study period, the mean of DTR from 2009 to 2013 was 8.05 °C (range, 1.10–17.6 °C) (Table 2). The daily average temperature was 12.41 °C (range, −14.5–31.8 °C); additionally, relative humidity (RH) was 60.1% (range, 20–97%). The mean concentrations of PM_10_ and O_3_ were 47.14 μg/m^3^ and 20.6 ppb, respectively.

**Fig. 1:**
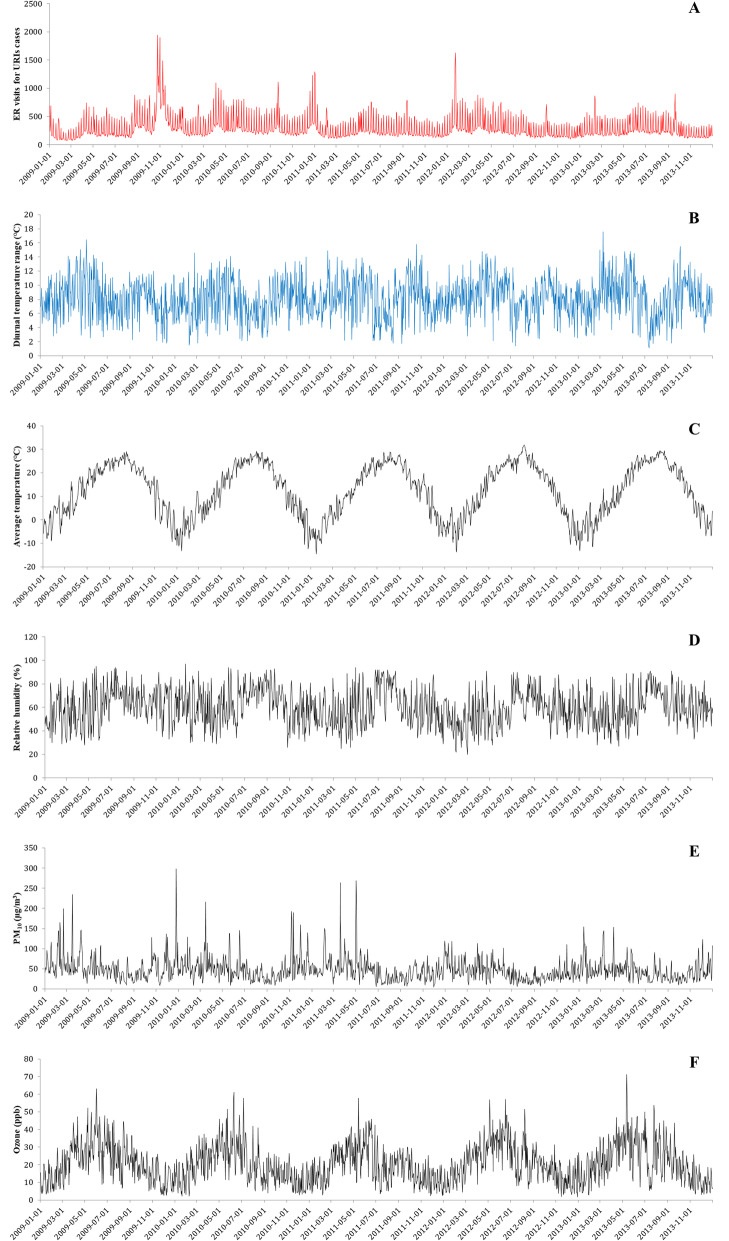
Time series plot of (**a**) emergency room (ER) visits for upper respiratory tract infections (URI) (number of daily cases), (**b**) diurnal temperature range (DTR), (**c**) average temperature, (**d**) relative humidity, (**e**) particulate matter with a median aerometric diameter < 10 microns (PM_10_), and (**f**) ozone (O_3_) in Seoul, Korea, 2009 to 2013

The number of daily ER visits for URI, DTR (°C), average temperature (°C), RH (%), PM_10_ (μg/m3), and O_3_ (ppb) during the study period are presented in Fig. 1. ER visits for URI varied seasonally, with a major peak in the winter months (December to February) and a minor peak in the spring (March to May).

**Fig. 2:**
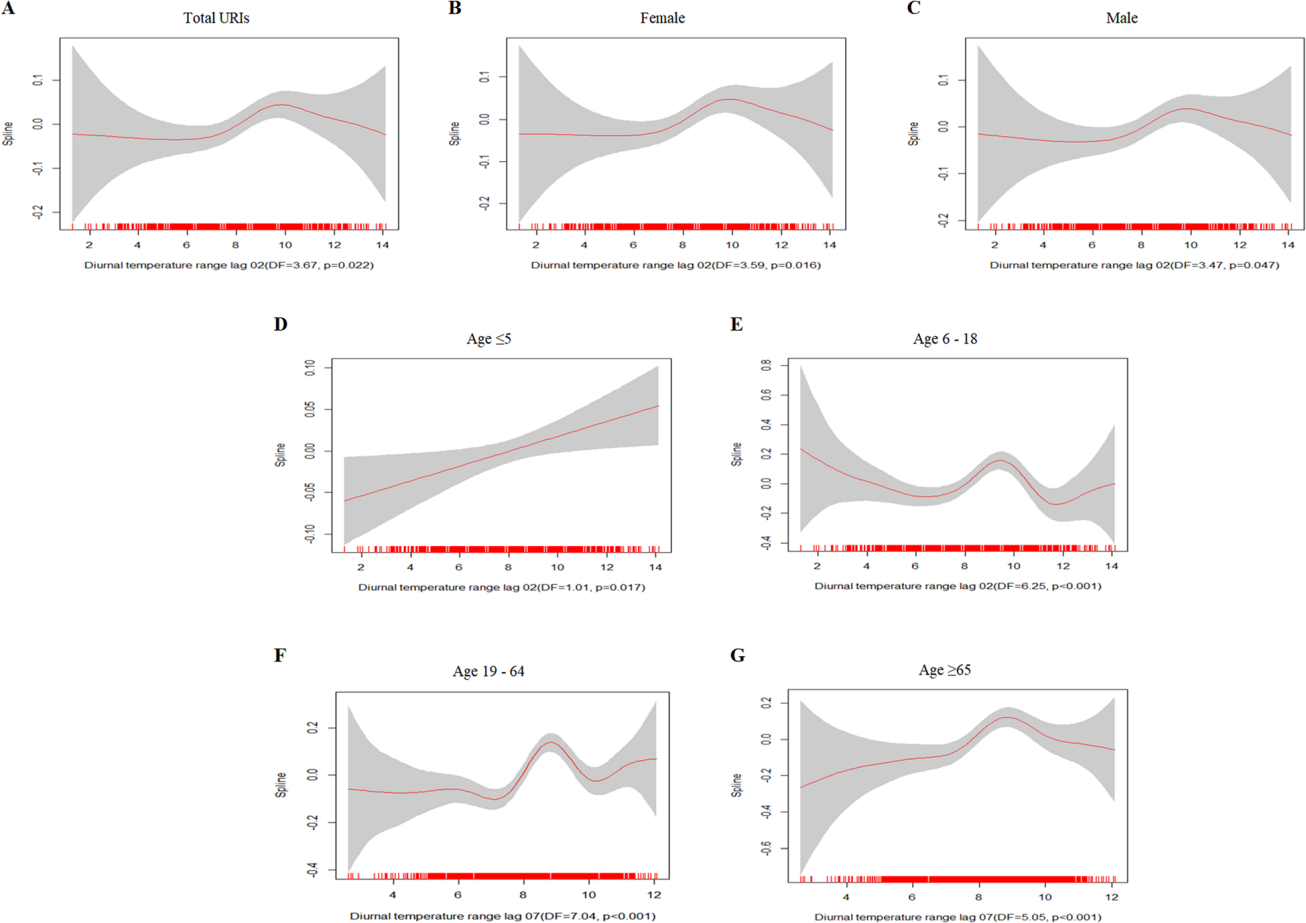
The smoothed exposure-response curves between diurnal temperature range (DTR) and emergency room (ER) visits for upper respiratory tract infections (URI) in different subgroups in Seoul, Korea, 2009 to 2013. The line represents a spline curve and the shaded area represents the 95% confidence intervals. The models were controlled for the average temperature, relative humidity, particulate matter with a median aerometric diameter < 10 microns (PM_10_), ozone (O_3_), time trend, seasonality, day of the week (DOW), and holiday

The smoothed exposure-response plots of DTR and ER visits for URI at the smallest AIC after controlling for average temperature, RH, PM_10_, O_3_, time trend, seasonality, DOW, and holiday are presented in Fig. 2. The plots demonstrate a clear nonlinear relationship, except under 5 years old. Overall, as DTR increased, the incidence of URI increased.

Table 3 shows the results of analyzing the piecewise regression model by finding the breakpoint value based on the results of the multivariate analysis. When the DTR (lag 02) was 6.57 °C to 11.03 °C, the percent change was significant at 6.01% (95% CI 2.45–9.69) for every 1 °C increase in youth subjects aged for 6 to 18 years.

**Table 3:** The effect of a 1 °C increase in diurnal temperature range on daily emergency room visits for acute upper respiratory tract infections (URI) in Seoul, Korea, 2009 to 2013

	Lag	Break point (°C)	Percent change	95% CI		***p*** value
Total URI	02	<6.57	0.84	−2.79	4.61	0.6481
		≥6.57	**1.42**	**0.04**	**2.82**	**0.0401**
Female	02	<6.57	0.56	−3.31	4.59	0.7746
		≥6.57	**1.54**	**0.07**	**3.03**	**0.0364**
Male	02	<6.57	1.05	−2.50	4.74	0.5583
		≥6.57	1.31	−0.05	2.69	0.0547
Age ≤ 5 years	02	<6.57	1.01	−2.07	4.18	0.5171
		≥6.57	**1.45**	**0.32**	**2.60**	**0.0103**
Age 6–18 years	02	<6.57	−2.32	−8.19	3.93	0.45
		6.57≤BP<11.03	**6.01**	**2.45**	**9.69**	**0.0006**
		≥11.03	8.55	−0.50	18.42	0.0593
Age 19–64 years	07	<6.73	−2.13	−7.79	3.88	0.47
		≥6.73	**2.77**	**0.39**	**5.20**	**0.0195**
Age ≥ 65 years	07	<6.73	2.48	−7.59	13.65	0.6357
		≥6.73	1.66	−1.83	5.28	0.3449

Bolded values indicate statistically significant differences (i.e., *p* < 0.05)

## Discussion

The results of our study demonstrated the short-term effect of DTR on ER visits for acute URI in Korea. After adjustment for potential confounding by time-trends, seasonality, DOW, public holidays, average temperature, RH, PM_10_, and O_3_, we found that the incidence of URI increased with higher DTR. Evidence accumulated in this study suggests that DTR was an important risk factor for URI.

In the multi-variable model that controlled for climate factors and air pollutants, the percentage change in ER visits for URI and their associations with DTR are summarized in Table S2. DTR was associated with ER visits for URI for all patients on lag 02 days. The cumulative day (lag 02) effect of DTR per 1 °C increment was associated with a 1.18% (95% CI 0.27–2.09) increase in total URI. In Cordoba, Argentina, hospital admissions for URI increased by 0.29% (95% CI 0.08–0.67%) at lag 0 [10]. In Seoul, Korea, hospital admissions for total respiratory disease increase by 1.3% (95% CI 0.3–2.4%) at lag 09 [11]. In Shanghai, China, ER visits for respiratory tract infections increased by 2.08% (95% CI 1.24–2.93%) at lag 01 [9].

In subgroup analyses for sex, the effect of DTR variation on the URI was similar for both female and male. The female group increased by 1.32% (95% CI 0.35–2.30%) at lag 02, and the male group increased by 1.05% (95% CI 0.16–1.95%) at lag 02. Otherwise, in Beijing, China, the ER admissions for total respiratory disease among males increased by 1.17% (95% CI 0.27–3.18%) at lag 07, but the female group did not exhibit a DTR effect [8].

In age-specific analysis, we found that children (≤ 5 years) were affected by DTR per 1 °C, with cumulative effect estimates (lag 02) of 0.90% (95% CI, 0.15–1.65%), adults (19–64 years) with 2.98% (95% CI 1.32–4.67%) at lag 07, and the elderly (≥ 65 years of age) with 3.63% (95% CI 1.08–6.25%) at lag 07; however, youth (6–18 years) were not affected (Figure S1). In Cordoba, Argentina, hospital admissions for URI increased in children (≤ 5 years of age) with a 0.52% (95% CI, 0.12–0.93%) and the elderly (≥ 60 years) with 1.14% (95% CI 0.41–1.93%) at lag 0. However, youth (6–18 years) and adults (19–59 years) were not significantly affected [10]. As illustrated in Figure S2, in the youth (6–18 years) group, there are certain temperature intervals with significant relative risk. When the DTR of lag 02 was 9.47 °C, the relative risk was 1.277 (95% limit 1.264–1.289). It can be seen that the low DTR distribution at lag 02 mainly occurs in the summer and winter, and the high DTR mainly occurs in the spring and autumn. In Fig. 2, the effect of DTR decreased and not significant when the DTR more than 10 °C. This is probably because the single day exceeds 10 °C are rare, and the cumulative day exceed 10 °C is more rare. Table S2 shows that the effects of cumulative day DTR are greater than the effects of any single day’s DTR. In age less than 5, the cumulative day 03 has the greatest effect on URI, while in the age over 19, the greater the cumulative day within a week, the greater the effect on the incidence of URI. It can be assumed that having different incubation periods of the pathogens can be a factor in explaining this lag effect of cumulative day DTR. The different distribution of causative pathogens by age may also explain the difference of lag by age. Another plausible explanation for the lag effect by age is the transmission characteristics of respiratory pathogen. Some respiratory viruses like influenza, make outbreaks in children, and then transmitted to adults [22]. However, the evidence to explain this lag effects should be explored in further studies.

Based on the results of the piecewise regression model (Table 3), the percent change of 6-18 years of age compared to other ages is presumed to be influenced by the group life that occurs at the beginning of the semester in spring and autumn. In this regard, DTR was associated with an ER visits for URI in all age groups.

The effect of DTR is also associated with respiratory syncytial virus (RSV), asthma, and chronic obstructive pulmonary disease (COPD). In Fukuoka, Japan, the number of RSV cases from 2006 to 2012 increased, with an RR of 3.30% (95% CI 1.62–6.60%) for a 1 °C increase at lag 0–16 weeks [23]. In Brisbane, Australia, ER admissions for asthma among the children (0–14 years of age) from 2003 to 2009, increased by 31% (95% CI 11–58%) of 15 °C versus 10 °C, at a moving average lag of 09 [24]. ER admissions for COPD between 2001 and 2002 in Taichung, Taiwan, increased by 13.9% (95% CI 0.8–28.8%) of ≥ 9.6 °C versus < 6.6 °C [25]. Also, the DTR was associated with stroke, COPD, and coronary artery-related death [26], and the morbidity of childhood pneumonia [27].

Results of the present study support a relationship between DTR and ER visits for URI. While the exact physiological mechanism(s) of effect of DTR on URI is no clear, previous study found that sudden temperature changes can cause inflammatory responses in nasal epithelial cells [28], which could lead to URI.

There were several limitations to the present study. First, data regarding ER visits for URI were from only one city, which may have introduced selection bias. In addition, although there was an increase in coverage for all hospitals in Seoul, there was no tendency to increase the number of cases by year. ER visits show a severe level, and small proportion of the total effects of DTR. And, because our raw data uses public data that cannot identify individuals, we could not identify re-visits for patients with URI from the available data. Second, we used outdoor monitoring data to represent personal exposure to DTR and other climate factors and air pollutants, which may have resulted in exposure misclassification(s). However, exposure measurement error was unavoidable, but has been shown to bias toward the null. It makes the association underestimated [29]. Third, some previous studies have found that particulate matter < 2.5 μm in aerodynamic diameter (PM_2.5_) is an adverse factor for URI [30, 31]. However, data regarding PM_2.5_ were unavailable during the study period; therefore, further studies need are warranted. And we could not consider seasonal allergies as a modifier due to lack of information. And further studies related to the weather app notices need to be conducted to prevent not only URI, which are affected by the DTR but also various health outcomes.

## Conclusions

In summary, our study found that DTR was associated with ER visits for URI in Seoul between 2009 and 2013. In addition, the results suggested that age is a modifier of the incidence of URI according to DTR.

## Supplementary Information

**Additional file 1: Table S1.** Spearman’s correlations among emergency room visits for upper respiratory tract infections, climate factors, and air pollutants in Seoul, Korea, 2009 to 2013.

**Additional file 2: Table S2.** Percent change in emergency room visits for acute upper respiratory tract infections (URI) associated with a 1°C increase in dirunal temperature range in Seoul, Korea, 2009 to 2013.

**Additional file 3: Figure S1.** Percentage change (%) in emergency room (ER) visits for upper respiratory tract infections (URI) in different subgroups associated with a 1°C increase in diurnal temperature range (DTR) in Seoul, Korea, 2009–2013. The X-axis shows the lag of DTR (°C) in the different subgroups. The Y-axis is the estimated percentage change (%). The circles represent the central estimate and the vertical lines the 95% confidence interval. The models were controlled for the average temperature, relative humidity, particulate matter with a median aerometric diameter < 10 microns (PM_10_), ozone (O_3_), time trend, seasonality, day of the week (DOW), and holidays.

**Additional file 4: Figure S2.** The relative risk (RR) between diurnal temperature range (DTR) and emergency room (ER) visits for upper respiratory tract infections (URI) in different subgroups in Seoul, Korea, 2009–2013. The X-axis shows the moving averages of DTR (°C). The Y-axis represents the estimated relative risk (RR). The line represents central estimates and the dotted lines represent the 95% upper and lower limits. The models were controlled for the average temperature, relative humidity, particulate matter with a median aerometric diameter < 10 microns (PM_10_), ozone (O_3_), time trends, seasonality, day of the week (DOW), and holidays.

## Data Availability

The data were obtained from the National Emergency Department Information System (NEDIS) of National Emergency Medical Center in Korea. Due to legal restrictions, the database cannot be made publicly available. However, data are available from the authors upon reasonable request and with permission of the National Emergency Medical Center.
