# Supplementary material for: Effect of diurnal temperature range on emergency room visits for acute upper respiratory tract infections
Source: Environ Health Prev Med. 2021 May 3;26:55. doi: 10.1186/s12199-021-00974-w (PMC8091143; doi:10.1186/s12199-021-00974-w)
Supplement: Supplementary file 1 — Additional file 1: Table S1. Spearman’s correlations among emergency room visits for upper respiratory tract infections, climate factors, and air pollutants in Seoul, Korea, 2009 to 2013. [file 12199_2021_974_MOESM1_ESM.docx]

**Table S1**. Spearman’s correlations among emergency room visits for upper respiratory tract infections, climate factors, and air pollutants in Seoul, Korea, 2009 to 2013.

| **Variable** | **URI** | **DTR** | **Tavr** | **RH** | **PM_10_** |
| --- | --- | --- | --- | --- | --- |
| **DTR** | **0.077*** | - |  |  |  |
| **Tavr** | **0.136*** | 0.025 | - |  |  |
| **RH** | 0.020 | -0.542 | **0.386*** | - |  |
| **PM_10_** | 0.005 | **0.255*** | **-0.284*** | **-0.170*** | - |
| **Ozone** | **0.205*** | **0.245*** | **0.379*** | **-0.207*** | -0.014 |

* Bolded values indicate statistically significant differences (ie, *p* < 0.001). URI: Acute upper respiratory tract infection; DTR: diurnal temperature range; Tavr: average temperature; RH: Relative humidity; PM_10_: particulate matter with a median aerometric diameter < 10 microns.
