# Supplementary material for: Effect of diurnal temperature range on emergency room visits for acute upper respiratory tract infections
Source: Environ Health Prev Med. 2021 May 3;26:55. doi: 10.1186/s12199-021-00974-w (PMC8091143; doi:10.1186/s12199-021-00974-w)
Supplement: Supplementary file 2 — Additional file 2: Table S2. Percent change in emergency room visits for acute upper respiratory tract infections (URI) associated with a 1°C increase in dirunal temperature range in Seoul, Korea, 2009 to 2013. [file 12199_2021_974_MOESM2_ESM.docx]

**Table S2.** Percent change in emergency room visits for acute upper respiratory tract infections (URIs) associated with a 1℃ increase in diurnal temperature range in Seoul, Korea, 2009 to 2013.

| **Lag** | **Total URIs** | **Female** | **Male** | **Age ≤ 5 years** | **Age 6–18 years** | **Age 19–64 years** | **Age ≥ 65 years** |
| --- | --- | --- | --- | --- | --- | --- | --- |
| **Single day** | | | | | | | |
| 0 | 0.60 (-0.04, 1.25) | 0.64 (-0.05, 1.32) | 0.57 (-0.06, 1.21) | **0.55 (0.02, 1.08)*** | 0.58 (-0.57, 1.75) | **0.87 (0.14, 1.62)*** | 1.05 (-0.08, 2.18) |
| 1 | **0.65 (0.01, 1.29)*** | **0.74 (0.06, 1.42)*** | 0.56 (-0.06, 1.19) | 0.49 (-0.04, 1.02) | 0.69 (-0.46, 1.85) | **0.98 (0.25, 1.72)*** | **1.83 (0.70, 2.96)*** |
| 2 | 0.49 (-0.16, 1.14) | 0.58 (-0.11, 1.27) | 0.41 (-0.23, 1.05) | 0.30 (-0.24, 0.83) | 0.71 (-0.45, 1.89) | **0.79 (0.04, 1.53)*** | 0.96 (-0.17, 2.09) |
| 3 | -0.03 (-0.70, 0.64) | -0.04 (-0.75, 0.68) | -0.03 (-0.69, 0.63) | -0.14 (-0.69, 0.42) | 0.04 (-1.16, 1.26) | 0.33 (-0.44, 1.11) | 0.18 (-0.97, 1.35) |
| 4 | -0.51 (-1.32, 0.31) | -0.60 (-1.46, 0.27) | -0.42 (-1.21, 0.39) | -0.35 (-1.04, 0.34) | -1.21 (-2.65, 0.25) | 0.06 (-0.88, 1.00) | 0.10 (-1.29, 1.52) |
| 5 | -0.08 (-0.77, 0.61) | 0.04 (-0.70, 0.78) | -0.18 (-0.86, 0.50) | -0.17 (-0.73, 0.41) | -0.44 (-1.67, 0.81) | 0.48 (-0.32, 1.28) | 0.58 (-0.62, 1.78) |
| 6 | 0.01 (-0.64, 0.65) | 0.11 (-0.58, 0.80) | -0.09 (-0.72, 0.55) | -0.26 (-0.79, 0.27) | -0.06 (-1.21, 1.11) | 0.60 (-0.15, 1.35) | 0.30 (-0.82, 1.44) |
| 7 | 0.22 (-0.44, 0.87) | 0.40 (-0.29, 1.10) | 0.05 (-0.59, 0.70) | 0.03 (-0.51, 0.57) | -0.12 (-1.29, 1.06) | **0.80 (0.05, 1.56)*** | 0.65 (-0.49, 1.79) |
| **Cumulative day** | | | | | | | |
| 01 | **0.99 (0.19, 1.80)*** | **1.09 (0.23, 1.95)*** | **0.90 (0.11, 1.69)*** | **0.82 (0.16, 1.48)*** | 1.00 (-0.44, 2.46) | **1.47 (0.54, 2.40)*** | **2.29 (0.87, 3.74)*** |
| 02 | **1.18 (0.27, 2.09)*** | **1.32 (0.35, 2.30)*** | **1.05 (0.15, 1.95)*** | **0.90 (0.15, 1.65)*** | 1.32 (-0.31, 2.98) | **1.78 (0.73, 2.84)**** | **2.64 (1.02, 4.29)*** |
| 03 | **1.09 (0.07, 2.12)*** | **1.23 (0.14, 2.32)*** | 0.98 (-0.02, 1.99) | 0.77 (-0.06, 1.62) | 1.26 (-0.57, 3.13) | **1.87 (0.69, 3.06)*** | **2.61 (0.80, 4.46)*** |
| 04 | 0.99 (-0.19, 2.18) | 1.09 (-0.16, 2.36) | 0.89 (-0.26, 2.06) | 0.71 (-0.26, 1.69) | 0.84 (-1.26, 2.98) | **2.00 (0.65, 3.38)*** | **2.89 (0.79, 5.03)*** |
| 05 | 0.96 (-0.34, 2.28) | 1.14 (-0.24, 2.54) | 0.81 (-0.46, 2.10) | 0.63 (-0.44, 1.71) | 0.60 (-1.72, 2.97) | **2.33 (0.83, 3.86)*** | **3.33 (1.01, 5.71)*** |
| 06 | 0.90 (-0.46, 2.29) | 1.14 (-0.31, 2.61) | 0.69 (-0.65, 2.05) | 0.42 (-0.71, 1.55) | 0.52 (-1.92, 3.02) | **2.61 (1.03, 4.22)**** | **3.36 (0.92, 5.85)*** |
| 07 | 0.99 (-0.43, 2.44) | 1.33 (-0.19, 2,87) | 0.69 (-0.71, 2.10) | 0.41 (-0.76, 1.59) | 0.42 (-2.12, 3.03) | **2.98 (1.32, 4.67)**** | **3.63 (1.08, 6.25)*** |

Data presented as percent change (95% confidence interval). *Bolded values indicate statistically significant differences (ie, *: *p* < 0.05, **: *p* < 0.001). Models were controlled for average temperature, relative humidity, particulate matter with a median aerometric diameter < 10 microns, ozone, time trend, seasonality, day of the week, and holidays.
