# Supplementary figures and images for: Effect of diurnal temperature range on emergency room visits for acute upper respiratory tract infections
Source: Environ Health Prev Med. 2021 May 3;26:55. doi: 10.1186/s12199-021-00974-w (PMC8091143; doi:10.1186/s12199-021-00974-w)

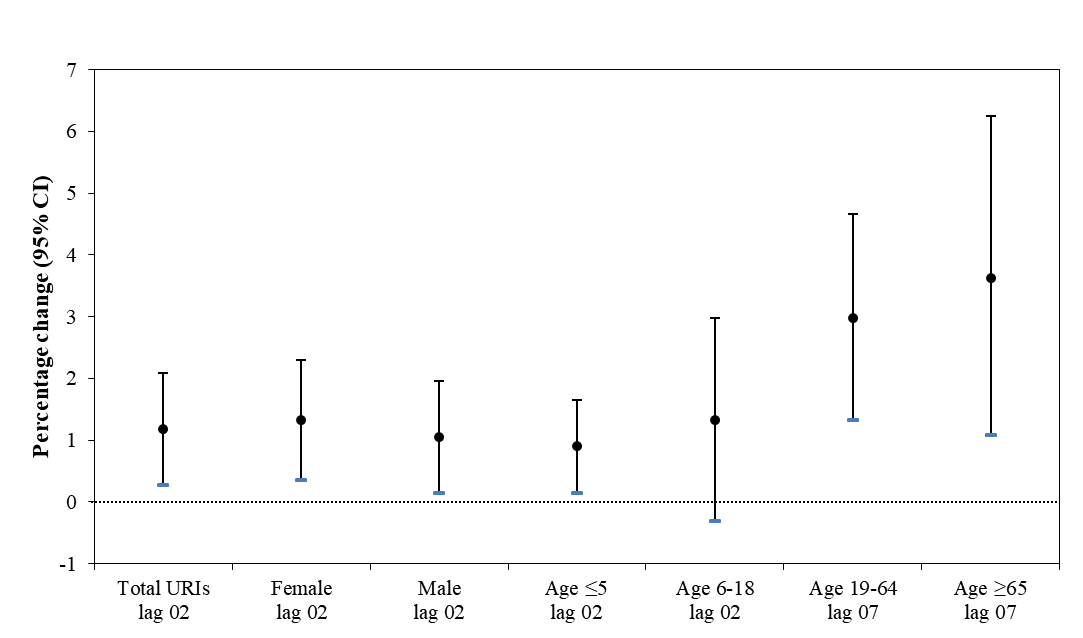

Supplement: Supplementary file 3 — Additional file 3: Figure S1. Percentage change (%) in emergency room (ER) visits for upper respiratory tract infections (URI) in different subgroups associated with a 1°C increase in diurnal temperature range (DTR) in Seoul, Korea, 2009–2013. The X-axis shows the lag of DTR (°C) in the different subgroups. The Y-axis is the estimated percentage change (%). The circles represent the central estimate and the vertical lines the 95% confidence interval. The models were controlled for the average temperature, relative humidity, particulate matter with a median aerometric diameter < 10 microns (PM10), ozone (O3), time trend, seasonality, day of the week (DOW), and holidays. [file 12199_2021_974_MOESM3_ESM.tiff]

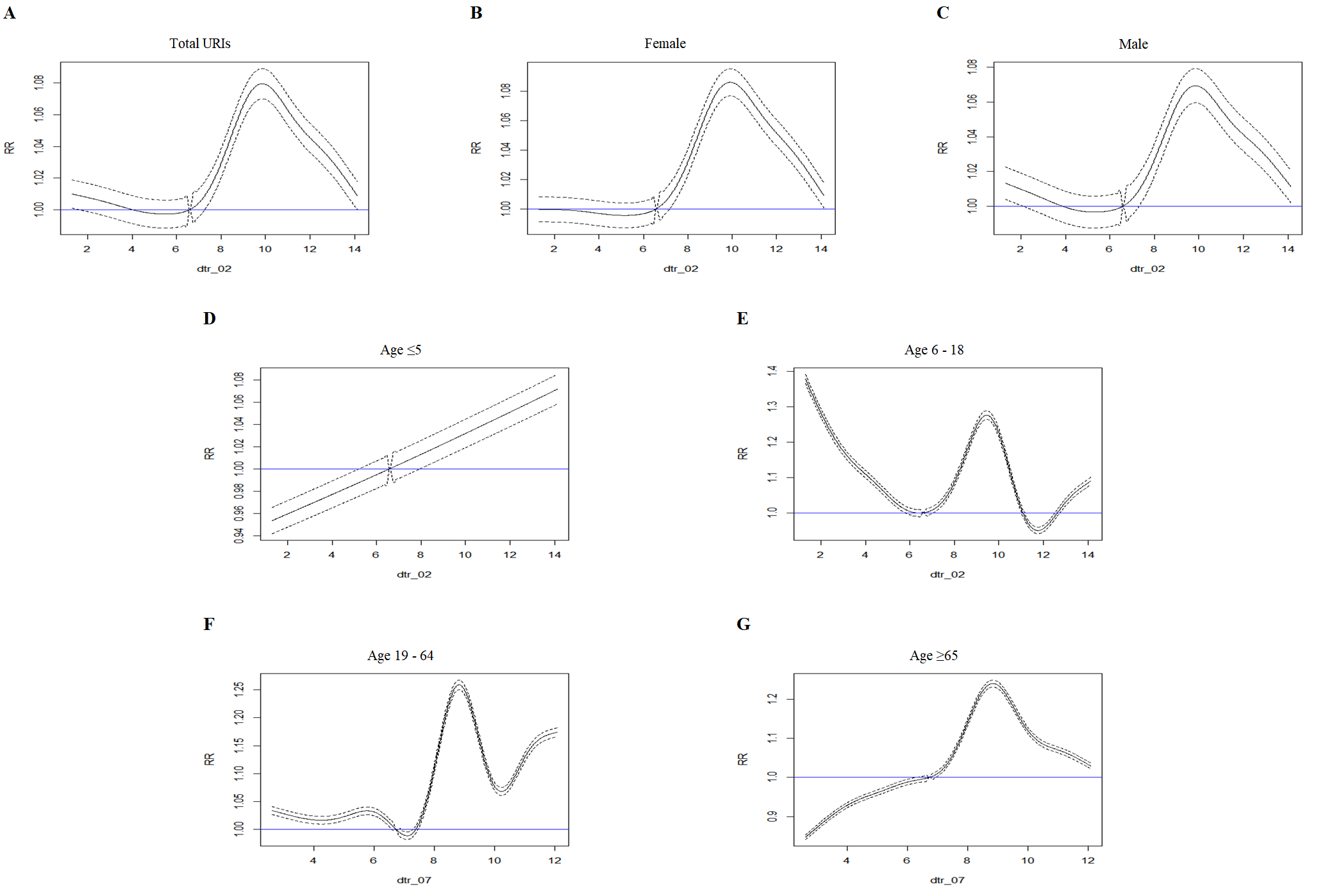

Supplement: Supplementary file 4 — Additional file 4: Figure S2. The relative risk (RR) between diurnal temperature range (DTR) and emergency room (ER) visits for upper respiratory tract infections (URI) in different subgroups in Seoul, Korea, 2009–2013. The X-axis shows the moving averages of DTR (°C). The Y-axis represents the estimated relative risk (RR). The line represents central estimates and the dotted lines represent the 95% upper and lower limits. The models were controlled for the average temperature, relative humidity, particulate matter with a median aerometric diameter < 10 microns (PM10), ozone (O3), time trends, seasonality, day of the week (DOW), and holidays. [file 12199_2021_974_MOESM4_ESM.tiff]
